# Exercise Performance as a Predictor for Balance Impairment in COPD Patients

**DOI:** 10.3390/medicina55050171

**Published:** 2019-05-20

**Authors:** Chalerm Liwsrisakun, Chaicharn Pothirat, Warawut Chaiwong, Chaiwat Bumroongkit, Athavudh Deesomchok, Theerakorn Theerakittikul, Atikun Limsukon, Pattraporn Tajarernmuang, Nittaya Phetsuk

**Affiliations:** Division of Pulmonary, Critical Care and Allergy, Department of Internal Medicine, Faculty of Medicine, Chiang Mai University, Chiang Mai 50200, Thailand; chalermliw@hotmail.com (C.L.); warawut.chai@cmu.ac.th (W.C.); cbumroon@gmail.com (C.B.); adeesomc@yahoo.co.th (A.D.); theerakorn15@yahoo.com (T.T.); atikun.limsukon@gmail.com (A.L.); pat_taj99@hotmail.com (P.T.); phetsukn@gmail.com (N.P.)

**Keywords:** balance, falls, chronic obstructive pulmonary disease, timed up and go test, exercise, six-minute walk distance

## Abstract

*Background and objective:* Six-minute walk test (6-MWT) is a widely used test for assessing exercise performance in chronic obstructive pulmonary (COPD). However, the association between reduced walking distance and balance impairment in COPD has not been directly investigated. Therefore, the aim of this study was to identify exercise performance as a predictor for balance impairment in COPD. *Materials and Methods:* The cross-sectional study was conducted at a single visit involving stable COPD patients in Maharaj Nakorn Chiang Mai Hospital, Chiang Mai, Thailand from November 2015 to October 2017. The 6-MWT was measured for in all subjects. The prognostic confounding factors were also collected for all subjects. Balance test was measured using the Berg Balance Scale (BBS) and the Timed Up and Go (TUG) test. A cut-off score of BBS < 46 and/or the TUG ≥ 13.5 s was classified as balance impairment. Multivariable logistic regressions were performed to identify the six-minute walk distance (6-MWD) as a predictor for balance impairment in COPD. *Results:* Of the 176 COPD subjects assessed for eligibility, 118 COPD patients were enrolled including 86 males (72.9%) with a mean age of 73.5 ± 8.1 years. Thirty-three (28.0%) cases were classified with a balance impairment. The 6-MWD < 300 m was the predictor of balance impairment in COPD with an adjusted risk ratio of 10.10 (95%CI; 2.87, 35.61, *p*-value < 0.001). *Conclusions:* The 6-MWT is not only useful for evaluation of exercise performance, but also for prediction of balance impairment in patients with COPD. Our study suggests that the 6-MWD < 300 m is an important risk factor for balance impairment in COPD.

## 1. Introduction

Chronic obstructive pulmonary disease (COPD) is a respiratory disease that results in progressive airflow limitation and respiratory distress and is one of the most important causes of death worldwide [1]. In primary pulmonary pathophysiology, COPD patients are recognized to also suffer from many non-respiratory manifestations including cardiovascular system [2], neurological system [3,4,5], musculoskeletal system [6,7], nutritional depletion, and malnutrition [8,9,10,11]. Decreased exercise capacity and physical activities have been also been demonstrated in COPD patients [3].

Falling has considerable negative consequences for older adults, especially in COPD [12,13]. Falling is not only associated with mortality and morbidity but is also linked to poorer overall functional status. Therefore, reducing the risk of falling by an improved understanding of COPD-related balance impairment is necessary [14]. Impaired balance is an important intrinsic risk factor for falling in COPD patients [6,12,13,15,16]. From our knowledge, there are many risk factors for falling including advanced age, muscle weakness, poor physical activity, poor nutritional status, depression, cognitive impairment, visual deficit, cardiovascular comorbidity, female gender, and history of falling in the previous year [6,13,14].

The six-minute walk test (6-MWT) is a widely used practical simple test for assessing exercise performance [17]. This test reflects the functional exercise level for daily physical activities [18]. In some clinical settings, the assessment of balance impairment using the Berg Balance Scale (BBS) and/or Timed Up and Go (TUG) test may be difficult due to the limitations of time and health professionals. Therefore, balance testing with only one test would be better. A previous study suggested that 6-WWT may be a useful tool to assess the risk of balance impairment in patients with COPD [14]. Thus, the aim of this study was to identify exercise performance using 6-MWT as a predictor for balance impairment in COPD.

## 2. Materials and Methods

### 2.1. Study Population

One hundred seventy-six COPD patients were screened at the Outpatient Chest Clinic of Maharaj Nakorn Chiang Mai Hospital, Chiang Mai, Thailand from November 2015 to October 2017. Recruitment criteria included: Patients over 60 years of age with a diagnosis of COPD based on post-bronchodilator (BD) ratio of forced expiratory volume in first second (FEV_1_)/forced vital capacity (FVC) < 0.7 [19], ex-smokers with a smoking history of more than 10 pack-years, no history of acute exacerbation (AE) for at least six weeks prior to the enrollment, and receiving standard pharmacological treatment for COPD. Patients meeting any of the following respiratory criteria were excluded: Current diagnosis of asthma, current active respiratory disorders other than COPD, e.g. lung cancer, tuberculosis, or other significantly abnormal chest radiography not associated with COPD (documented within the past year). Those with specific disease states such as old cerebrovascular accident (CVA), neuromuscular diseases, and musculoskeletal disorders that affected walking were also excluded.

### 2.2. Study Procedures

The cross-sectional study was conducted at a single visit involving stable COPD patients in Maharaj Nakorn Chiang Mai Hospital. The balance test was measured using the BBS and the TUG test. The clinically relevant data (demographics, comorbidity, lung function test, severity classification of COPD, long-term oxygen therapy (LTOT), history of falling in the previous year, history of acute exacerbation of COPD (AECOPD), Health Related Quality of Life (HRQoL) using the St. George’s Respiratory Questionnaire (SGRQ) and COPD Assessment Test (CAT), severity of dyspnea using modified Medical Research Council (mMRC), visual deficits, six-minute walk distance (6-MWD), and Hospital Anxiety and Depression Scale (HADS)) were collected for all patients. The study was approved by the Ethics Committee of the Faculty of Medicine, Chiang Mai University [Institutional Review Board (IRB) approval number: MED-2558-03253, date of approval: 12 October 2015 and filed under Thai Clinical Trials Registry (Study ID: TCTR20151015001, date of approval: 15 October 2015). Written informed consent was obtained from each patient prior to the study.

### 2.3. Balance Test

The BBS was performed after the patients completed the questionnaire and lung function test. The BBS is a 14-item scale for assessment of functional activities in daily life tasks and is considered the gold standard test for static and dynamic balance abilities [20]. These activities are classified from 0 (unable) to 4 (independent). The sum of these points can reach a maximum of 56 points, and less points indicate a greater danger to the individual’s stability. A cut-off score of <46 of BBS can be successfully used to identify those who are at risk of falling [20,21]. The TUG test is a test of general mobility [22]. An armchair of standard height was used and a well-seen marking tape was placed 3 m away on the floor. The starting position was sitting with the patient’s back and hands resting on the back and arms of the chair, respectively. The participants were asked to stand up and walk toward the marker before crossing it, then return to their seats again. All subjects were instructed to perform the TUG test at their regular speed. The timing of the TUG started when the participant’s back came off from the back of the chair, and stopped when their buttocks and backs touched the seats and backs of the chair again, respectively. The TUG test was performed three times with a pause between repetitions with the shortest time being selected. The previous study suggested that TUG time ≥ 13.5 s was classified as balance impairment [23]. Due to acceptable sensitivity (91% and 80%) and specificity (82% and 100%) for the Berg Balance Scale and the TUG test for predicting risk of falling [21,23], a combined cut-off score of BBS < 46 and/or the TUG of ≥ 13.5 s was defined as balance impairment in our study. 

### 2.4. Exercise Performance

The 6-MWT is a simple practical test measuring the distance that subjects can quickly walk in a period of six minutes. It evaluates the global and integrated responses of all the systems involved during exercise, including the cardiopulmonary, systemic, and peripheral circulation, and neuromuscular metabolism [17]. The submaximal level of functional capacity can be assessed using the self-paced 6-MWT, as most of activities in daily living are performed at a submaximal level of exertion. Therefore, the American Thoracic Society (ATS) suggested that the 6-MWD could better reflect the functional exercise level for daily physical activities. In this study, the exercise performance was determined using a 6-MWT according to the guidelines of the ATS [17] in all subjects and the 6-MWD was recorded. 

### 2.5. Health-Related Quality of Life (HRQoL) and Dyspnea Severity

The HRQoL was assessed in all subjects using the Thai version of the SGRQ and Northern Thai dialect version of CAT [24]. The score ranged from 0–100 and 0–40 for the SGRQ and CAT, respectively. A higher score reflected a poorer HRQoL. The severity of dyspnea was also assessed in all subjects using the mMRC dyspnea scale [25]. The score ranged from 0–4 with the higher score representing more dyspnea.

### 2.6. Pulmonary Function Test

All subjects were evaluated for FVC, FEV_1_, and ratio of FEV_1_/FVC using a spirometer (Spiro Master PC, Chest M.I., Inc., Japan) following the American Thoracic Society (ATS)/European Respiratory Society (ERS) standard guidelines [26]. Values were calculated using Knudson’s reference equations [27]. However, for Asians, a correction factor of 0.94 was applied to the predicted FVC and FEV_1_ [28].

### 2.7. History of Falling 

History of falls and serious injuries from falls in the previous year were recorded using the Elderly Falls Screening Test (EFST) [29].

### 2.8. Visual Acuity Test

The visual acuity was evaluated in all subjects using the Snellen chart according to the standardized measurement of visual acuity [30]. Visual impairment in elderly was defined as a best corrected visual acuity of less than 20/40 [31].

### 2.9. Depression and Anxiety

Depression and anxiety were evaluated in all subjects using the Thai version of the Hospital Anxiety and Depression Scale (HADS). A score ≥ 11 indicated anxiety or depression [32].

### 2.10. Sample Size Calculation

Sample size calculation for multiple regression study was based on the previous study using G*Power program [33]. Five predictors in the model explained the fall risk. Therefore, 117 subjects needed to be studied to reject the null hypothesis that the power was 0.8 with an assumption of statistical significance at 0.05. 

### 2.11. Statistical Analysis

Results for numerical data were expressed as mean ± standard deviation (SD) or median, interquartile range (IQR) and those for categorical data were expressed as frequencies and percentages. Independent sample t-tests and Mann-Whitney U Test were used to compare differences between the impaired balance and nonimpaired balance groups for normally distributed and not normally distributed data, respectively. Fisher’s exact test was used to compare the proportional categorical data. Multivariable logistic regressions were performed to identify the exercise performance as a predictor for balance impairment in COPD when adjusted for other possible confounding factors from the previous findings [6,13,14] including age, female sex, cardiovascular co-morbidity, visual impairment, history of falling in the previous year, BMI, depression, and hypoxemia. Results were displayed as adjusted risk ratio (RR) together with a 95% confidence interval (CI) for RR. A *p*-value < 0.05 was considered as statistically significant. All statistical analyses were performed using STATA version 15 (StataCorp, College Station, TX, USA).

## 3. Results

**Figure 1:**
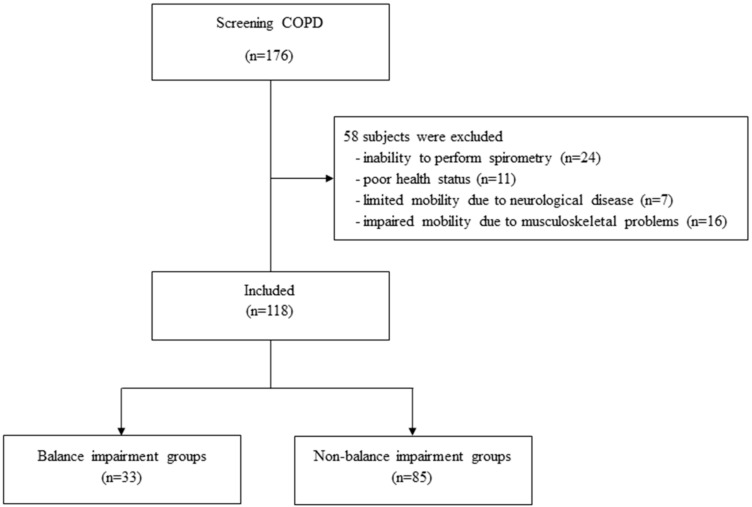
Flowchart describing the study population.

Of the 176 COPD subjects assessed for eligibility, 58 subjects were excluded due to: Inability to perform spirometry for a well-established diagnosis of COPD (n = 24), poor health status (n = 11), limited mobility due to neurological disease (n = 7), and impaired mobility due to musculoskeletal problems (n = 16) (Figure 1). A total of 118 COPD patients were included, 86 of which were males (72.9%) with a mean age of 73.5 ± 8.1 years. Thirty-three (28.0%) cases were classified with balance impairment and six (5.1%) cases had a history of falling in the previous year. According to severity of the disease, 32 (27.1), 34 (28.8), 5 (4.2), and 47 (39.8) cases were classified with the Global Initiative for Chronic Obstructive Lung Disease (GOLD) stages A, B, C, and D, respectively. The baseline characteristics of the balance impairment and non-balance impairment groups are shown in Table 1. There were no significant differences between the BMI, ratio of FE_1_/FVC, % predicted of FEV_1_, history of falling in the previous year, anxiety, depression, GOLD stage, and inhaled medications of subjects with the balance impairment and non-balance impairment groups. However, age, SGRQ score, CAT score, mMRC score, and TUG time of balance impairment group were significantly higher than the non-balance impairment groups. The balance impairment group had significantly higher frequency of females, single status, cardiovascular comorbidity, and hypoxemia. Furthermore, the balance impairment group had significantly lower % predicted of FVC, BBS score and exercise performance measured by the 6-MWT. Reliability of TUG test was calculated across the three trails using the intraclass correlation coefficients (ICC). Excellent ICC values were found for the TUG test (range from 0.967–0.990).

After adjustment for the possible confounding factors including age, female, cardiovascular co-morbidity, visual impairment, history of falling in the previous year, BMI, depression, and hypoxemia, multivariable analysis showed that the 6-MWD < 300 m was the only predictor of balance impairment in COPD patients with adjusted RR of 10.10 (95%CI; 2.87, 35.61, *p*-value < 0.001) (Table 2).

## 4. Discussion

The aims of this study were to identify exercise performance as a predictor for balance impairment in COPD. The main finding showed that the 6-MWD < 300 m was an important risk factor for balance impairment in COPD. 

Our study showed longer TUG times and lower BBS scores in the impaired balance group. The TUG times were 7.1 s longer and the BBS scores were 13.5 lower in the impaired balance group, indicative of clinically relevant deficits in measures of balance and functional mobility in patients with COPD. One previous study also demonstrated that an abnormal TUG time correlated with worse health outcomes [34]. However, the cut-off TUG time in that study was shorter than in our study (11.2 s vs. 13.5 s).

Nowadays, the BBS and the TUG tests are valid and widely used for assessing balance impairment in COPD [6,13,20,35]. However, multiple tests may have limitations in some clinics, e.g. crowding of patients, time consuming, and the limited number of healthcare professionals. Thus, a single simple test which could provide both exercise performance and balance impairment evaluation would be better.

The 6-MWT is a simple, widely validated, and reliable tool for assessing functional exercise capacity in COPD patients [36,37,38]. However, the association between reduced walking distance and increased fall risk or balance impairment in older COPD subjects has not been directly investigated. Our results showed the 6-MWD in the impaired balance group was 178 m shorter when compared to the nonimpaired balance group, indicative of clinically relevant deficits in measures of exercise performance in patients with impaired balance. The 6-MWD in our study was different from the previous findings which ranged from 49.0–111.0 m [13,14]. This distinction may be due to the differences in the TUG time between non-impaired and impaired balance groups (7.1 vs. 1.3–3.0 s in our study and the previous study, respectively) [13,14]. Moreover, this study showed strong associations between impaired balance and poor exercise capacity (6-MWD < 300 m). Depending on the level of impairment, the 6-MWT provided good estimates of exercise capacity [39] and overall functional performance [40]. For example, 6-MWD was proved to correlate with peak oxygen consumption in people with COPD [41] and impairment of lower limbs muscle function, including strength and power, were associated with falling risk in older adults [42]. Given these associations, it is likely that the shorter walking distance is correlated with the higher risk of balance impairment. 

This study has strengths that should be considered. First, this is the first reported study that identifies the exercise performance as a predictor for balance impairment in COPD. Second, many prognostic confounding factors including age, female gender, cardiovascular co-morbidity, visual impairment, history of falling in the previous year, BMI, depression, and hypoxemia were included for the multivariable analysis. However, this study has some limitations. First, a healthy control group was not included in this study. Therefore, age-, sex-matched controls should be included to determine if these factors differ in healthy elderly individuals. Second, the important risk factors for falling or balance impairment in the elderly such as postural control, muscle strength, cognitive function, and physical activity level were not included for the multivariable analysis. 

## 5. Conclusions

The 6-min walk test not only evaluates the exercise ability but also predicts the risk of balance impairment in patients with COPD. This finding suggests that 6-MWD < 300 m is an important risk factor for balance impairment in COPD. 

## Figures and Tables

**Table 1:** Baseline and clinical characteristics of participants.

Characteristics	Total Patients (N = 118)	Impaired Balance (N = 33)	Non-Impaired Balance (N = 85)	*p*-Value
Age (Years)	73.5 ± 8.1	78.8 ± 6.9	71.4 ± 7.6	<0.001
Male gender	86 (72.9)	15 (45.5)	71 (83.5)	<0.001
BMI (kg/m^2^)	21.1 ± 4.4	20.2 ± 5.1	21.5 ± 4.1	0.215
Marital status				0.036
Single/Widow	32 (27.1)	14 (42.4)	18 (21.2)	
Married	86 (72.9)	19 (57.6)	67 (78.8)	
Spirometric results (Median, IQR)				
FEV_1_/FVC (%)	56.0 (47.8–62.3)	59.3 (48.0–64.1)	54.5 (47.3–62.1)	0.248
% Predicted of FEV_1_	59.7 (42.3–69.9)	58.0 (37.1–70.4)	60.0 (44.1–69.5)	0.755
% Predicted of FVC	81.4 (65.9–98.5)	79.1 (57.0–87.8)	84.3 (69.0–100.2)	0.086
Cardiovascular co-morbidity	35 (29.7)	16 (48.5)	19 (22.4)	0.007
Impaired Visualization	12 (10.2)	5 (15.2)	7 (8.2)	0.312
History of falling in the previous year	6 (5.1)	4 (12.1)	2 (2.4)	0.051
History of AECOPD in the previous year	28 (23.7)	10 (30.3)	18 (21.2)	0.338
Hypoxemia	13 (11.0)	7 (21.2)	6 (7.1)	0.045
Anxiety	3 (2.5)	1 (3.2)	2 (2.4)	0.834
Depression	10 (8.5)	5 (15.2)	5 (5.9)	0.140
SGRQ (Median, IQR)	38.4 (22.3–53.2)	44.3 (34.2–54.9)	37.7 (16.9–53.5)	0.063
CAT (Median, IQR)	11.0 (6.0–16.0)	13.0 (9.5–19.5)	9.0 (5.0–15.0)	0.002
MMRC (Median, IQR)	2.0 (1.0–3.0)	3.0 (2.0–3.0)	2.0 (1.0–2.0)	<0.001
TUG (Seconds) (Median, IQR)	10.1 (7.9–12.6)	14.8 (13.5–18.6)	8.9 (7.5–10.5)	<0.001
BBS	48.85 ± 7.59	39.15 ± 7.65	52.61 ± 2.58	<0.001
6-MWD (Meters) (Median, IQR)	316.0 (217.3–405.0)	150.0 (85.0–239.0)	353.0 (300.0–426.5)	<0.001
GOLD Classification				0.107
A	32 (27.1)	4 (12.1)	28 (32.9)	
B	34 (28.8)	13 (39.4)	21 (24.7)	
C	5 (4.2)	1 (3.0)	4 (4.7)	
D	47 (39.8)	15 (45.5)	32 (37.7)	
Inhaled Medications				0.762
SABA	1 (0.8)	0 (0)	1 (1.2)	
LABA	14 (11.9)	5 (15.1)	9 (10.6)	
LAMA	10 (8.4)	2 (6.1)	8 (9.4)	
ICS + LABA	46 (39.0)	11 (33.3)	35 (41.2)	
ICS + LABA + LAMA	47 (39.8)	15 (45.5)	32 (37.7)	

6-MWD, six-minute walk distance; AECOPD, acute exacerbation of chronic obstructive pulmonary disease; BBS, Berg Balance Scale; BMI, body mass index; CAT, COPD Assessment Test; FEV_1_, forced expiratory volume in first second; FVC, forced vital capacity; GOLD, Global Initiative for Chronic Obstructive Lung Disease; ICS, inhaled corticosteroids; IQR, interquartile range; LABA, long acting beta2-agonists; LAMA, long acting muscarinic antagonists; mMRC, modified Medical Research Council; SABA, short acting beta2-agonists; SGRQ, St. George’s Respiratory Questionnaire; TUG, Timed Up and Go.

**Table 2:** Multivariable analysis for identifying predictors of balance Impairment in chronic obstructive pulmonary (COPD) patients.

Risk Factors	Adjusted Risk Ratio	95% CI	*p*-Value
6-MWD < 300 m	10.10	2.87–35.61	<0.001
Age ≥ 70 years	1.95	0.71–5.30	0.193
Female gender	1.71	0.78–3.74	0.181
Cardiovascular co-morbidity	1.18	0.51–2.71	0.700
Impaired visualization	1.16	0.41–3.26	0.778
History of falling in the previous year	1.11	0.34–3.62	0.858
BMI < 18.5 kg/m^2^	1.05	0.47–2.34	0.901
Marital status: married	0.97	0.45–2.07	0.936
Depression	0.87	0.31–2.47	0.798
Hypoxemia	0.83	0.26–2.58	0.741

6-MWD, six-minute walk distance; BMI, body mass index; CI, confidence interval.
